# Spirometric Lung Function Among Smokers and Non-Smokers: A Cross-Sectional Study in University Students

**DOI:** 10.3390/ijerph23060709

**Published:** 2026-05-27

**Authors:** Khaldoun Tabbah, Abdulrahman Salem Abufanas, Ahmad Jalal Kanawati, Safielrahman Haitham Sami Elawaddlly, Dena Nashaat Hamza, Mohamad Mohamad Munzer Madarati, Abdul Ilah Ghazwan Dakak, Doha Farouk Abdelhafiz, Mahmoud Tariq Al Ammour

**Affiliations:** 1 Clinical & Medical Education Department, College of Medicine, Ajman University, Ajman P.O. Box 346, United Arab Emirates; 2 Center of Medical and Bio-Allied Health Sciences Research, Ajman University, Ajman P.O. Box 346, United Arab Emirates; 3 College of Medicine, Ajman University, Ajman P.O. Box 346, United Arab Emirates; 201910664@ajmanuni.ac.ae (A.S.A.); 202010539@ajmanuni.ac.ae (S.H.S.E.);

**Keywords:** e-cigarettes, cigarettes, shisha, lung function, spirometry, university students, FEV_1_/FVC ratio, MENA region, public health

## Abstract

**Highlights:**

**Public health relevance—How does this work relate to a public health issue?**

- This study relates to a major public health issue by showing that smoking, BMI, and sex are associated with early measurable changes in lung function among young adults.
- The study highlights the importance of early identification and prevention of respiratory risk factors to reduce the future burden of chronic respiratory diseases such as COPD.

**Public health significance—Why is this work of significance to public health?**

- This work is significant to public health because it identifies early, subclinical respiratory changes associated with smoking and BMI in a young population, before the development of overt disease.
- The study provides evidence to support targeted prevention strategies and early screening to reduce the future burden of chronic respiratory and non-communicable diseases.

**Public health implications—What are the key implications or messages for practitioners, policy makers, and/or researchers in public health?**

- This study implies that public health practitioners and policymakers should prioritize early smoking prevention and cessation programs, especially among university-aged males who show higher prevalence and worse lung function outcomes.
- The study also suggests that integrating lung health screening with broader lifestyle interventions (such as weight management) and encouraging further research on early respiratory changes can help reduce the future burden of chronic respiratory disease.

**Abstract:**

Background: With the increasing use of cigarettes and electronic nicotine delivery systems (ENDS) in young adults, growing concern exists regarding lung health among university students. While the adverse respiratory effects of smoking are well established in older populations, early functional changes among young adults remain less well studied. Identifying such changes in this vulnerable population is crucial due to the risk of detrimental long-term health effects and the role of implementing early preventive measures. This study aims to compare the effects of nicotine use, sex, and body mass index (BMI) on the spirometric lung function parameters, including FEV_1_, FVC, and FEV_1_/FVC ratio, of smokers (including cigarette, ENDS, shisha, and midwakh users) and non-smokers in a university population. Methods: This cross-sectional study was conducted at Ajman University, United Arab Emirates. A convenience sample of 652 smokers and non-smokers students was initially recruited voluntarily, of whom 630 participants met the spirometry acceptability criteria and were included in the final analysis. Lung function was assessed using spirometry performed according to the guidelines of the American Thoracic Society and European Respiratory Society. Forced expiratory volume in one second (FEV_1_), forced vital capacity (FVC), and the FEV_1_/FVC ratio were recorded and expressed as percentages of predicted values based on Global Lung Function Initiative reference equations. Lung function parameters were compared according to smoking status, sex, and BMI. Results: A total of 630 students were included (60.5% males; the majority aged 20–22 years). The prevalence of smoking was 28.9% and was significantly higher among males than females (38.6% vs. 14.1%; OR = 3.86, *p* < 0.001). Smokers demonstrated a significantly lower FEV_1_/FVC ratio compared with non-smokers (0.84 ± 0.07 vs. 0.86 ± 0.07, *p* < 0.001), despite slightly higher predicted FEV_1_ and FVC values. Males exhibited higher predicted lung volumes than females, whereas females had higher FEV_1_/FVC ratios (*p* < 0.001). Lung function varied significantly across BMI categories (*p* < 0.001), with increasing BMI associated with higher predicted lung volumes but lower FEV_1_/FVC ratios. Stratified analysis showed that male smokers had the lowest FEV_1_/FVC ratios, while female non-smokers had the highest. Conclusions: Smoking, sex, and BMI significantly influenced lung function in this cohort. Smokers demonstrated reduced FEV_1_/FVC ratios, indicating early airflow limitation. Males were more likely to smoke and had higher lung volumes, while females showed higher FEV_1_/FVC ratios. Increasing BMI was associated with higher lung volumes but lower FEV_1_/FVC ratios. These findings suggest that early pulmonary changes may occur in young adults, highlighting the importance of early screening, careful interpretation of spirometry, and the implementation of targeted public health interventions to reduce nicotine use, promote smoking cessation, and support lung health among university students.

## 1. Introduction

### 1.1. Burden of Cigarette Smoking and Electronic Nicotine Delivery System Use in Young Adults

Cigarette smoking remains a major global public health concern because of its well-established adverse effects on respiratory health and its contribution to preventable morbidity and mortality [1]. Smoking has also been associated with increased healthcare utilization and respiratory disease burden, particularly when combined with environmental and socioeconomic risk factors [2]. This impact is particularly concerning among university students, as young adults often underestimate the long-term health risks of tobacco use and may establish nicotine dependence during this formative stage of life [3]. Numerous studies have demonstrated that cigarette smoking negatively affects pulmonary function, commonly assessed using spirometry. Declines in key spirometric indices, including forced expiratory volume in 1 s (FEV_1_), forced vital capacity (FVC), and the FEV_1_/FVC ratio, have been consistently reported among smokers [4,5,6]. For example, a study among undergraduate students in Saudi Arabia showed an inverse relationship between smoking duration and spirometric parameters, with greater impairment observed in male smokers [4].

In recent years, the use of electronic nicotine delivery systems (ENDS), commonly known as e-cigarettes, has increased substantially, particularly among young adults [7]. These devices are often perceived as safer alternatives to conventional cigarettes [8]; however, emerging evidence indicates that e-cigarettes are not without health risks. Inhalation of e-cigarette aerosol has been linked to airway irritation, inflammatory responses, oxidative stress, and other adverse respiratory effects [9]. A study done among Chinese college students found no statistically significant difference in lung functions between e-cigarette smokers and non-smokers [10]. Nevertheless, long-term consequences of e-cigarette use remain incompletely understood, accumulating evidence suggests that prolonged exposure may contribute to airway dysfunction and exacerbate underlying pulmonary conditions [11].

### 1.2. Effects of Sex and Body Mass Index on Pulmonary Functions

Research has consistently shown that males generally have larger lungs and higher lung volumes, including higher forced vital capacity (FVC) and higher forced expiratory volume in 1 s (FEV_1_) [12,13]. However, interestingly, females have shown to have a higher ratio of FEV_1_/FVC [14]. When comparing the sex-based differences of nicotine use effect on pulmonary functions, a paper found that female lifetime nonsmokers had greater mean percentage predicted lung function values than male lifetime nonsmokers. On the other hand, female cigarette smokers had lower values than their male counterpart [15].

When it comes to Body Mass Index (BMI), higher BMI and weight gain were associated with declines in both FEV_1_ and FVC. Obesity also accelerated deterioration of lung function over time. Interestingly, FVC initially increased with weight gain in those thinnest, but subsequently decreased overtime [16].

### 1.3. The Role of Spirometry in Detecting Early Respiratory Effects of Nicotine Use

Spirometry is widely recognized as a reliable, non-invasive method for assessing pulmonary function and detecting early airflow limitation. Key spirometric parameters including FEV_1_, FVC, and FEV_1_/FVC ratio provide valuable information about airway obstruction and lung capacity. Large epidemiological studies have demonstrated the prognostic significance of these measures. For instance, the Baltimore Longitudinal Study of Aging reported that FEV_1_, after adjustment for age and smoking status, was a strong predictor of mortality [17]. Similarly, studies such as the National Health and Nutrition Examination Survey III (NHANES III) have demonstrated that a substantial proportion of asymptomatic smokers already exhibit reduced lung function, emphasizing the role of spirometry in detection of early respiratory impairment even before the onset of clinical symptoms [18]. The continued relevance of spirometry as a fundamental diagnostic tool in respiratory medicine has been highlighted in recent reviews [19].

### 1.4. Current Knowledge, Gaps in Existing Research, Study Objective and Significance

Although numerous studies have evaluated the effects of cigarette smoking on pulmonary function, data examining the combined influence of nicotine use, sex, and body mass index (BMI) on spirometric parameters among university students in the Middle East remain limited. Existing evidence suggests that even young and asymptomatic smokers may exhibit early alterations in pulmonary function before the development of overt respiratory disease [3,20]. Several studies have reported reductions in spirometric indices among cigarette smokers and waterpipe users, including university populations in the Middle East. For example, a study conducted among Palestinian university students demonstrated that waterpipe smoking was associated with adverse changes in spirometric parameters, emphasizing that alternative forms of tobacco use may also contribute to impaired respiratory function [21]. Similarly, emerging evidence regarding electronic nicotine delivery systems (ENDS) suggests that vaping may negatively affect respiratory health through airway irritation, oxidative stress, and inflammatory responses, although the long-term pulmonary consequences remain incompletely understood [9,10,11].

Despite increasing concern regarding vaping and alternative nicotine delivery systems among young adults, relatively few studies have comprehensively compared smokers and non-smokers while simultaneously evaluating the influence of sex and BMI on lung function within the same university population. Furthermore, most available studies have focused primarily on conventional cigarette smoking, whereas data addressing mixed nicotine exposure patterns—including ENDS, shisha, and midwakh use—remain scarce, particularly in Gulf-region university populations. Furthermore, relatively few studies have directly compared pulmonary function between users of electronic nicotine delivery systems (ENDS), conventional cigarette smokers, and non-smokers within the same university population, particularly while accounting for the potential influence of sex and BMI on spirometric outcomes. In addition, sex-related differences in spirometric responses to smoking and the influence of BMI on pulmonary function among young adults remain insufficiently explored in regional studies.

This study represents the second phase of a two-part investigation conducted among students at Ajman University. The first phase assessed the prevalence and patterns of nicotine product use, including conventional cigarettes and electronic nicotine delivery systems, in the same population. Phase I of our research demonstrated that students often initiate cigarette and e-cigarette use at an early age and may already exhibit signs of nicotine dependence [22]. Building on these findings, the present phase focuses on the objective assessment of pulmonary function to evaluate early physiological effects associated with smoking and vaping. Importantly, this study compared smokers—including users of cigarettes, ENDS, shisha, and midwakh—with non-smokers to determine whether nicotine exposure is associated with measurable changes in lung function.

Therefore, the present Phase II study aimed to evaluate the effects of nicotine use, sex, and body mass index (BMI) on spirometric lung function parameters, including FEV_1_, FVC, and FEV_1_/FVC ratio, among university students. By identifying potential early airflow alterations associated with nicotine exposure in this young population, the study provides regional evidence that may support preventive public health strategies, smoking cessation initiatives, and early respiratory screening programs targeting both traditional and alternative nicotine delivery systems among university students.

## 2. Materials and Methods

### 2.1. Study Design and Population

This cross-sectional study was conducted at Ajman University across all colleges to evaluate and compare pulmonary function among university students using spirometry. The study period was from 17 September 2023 to 30 November 2024 (around 1 year and 2 months). The inclusion criteria included any Ajman University student who was enrolled during the period of this study, while the exclusion criteria included all Ajman University faculty and staff.

### 2.2. Study Sample

**Figure 1:**
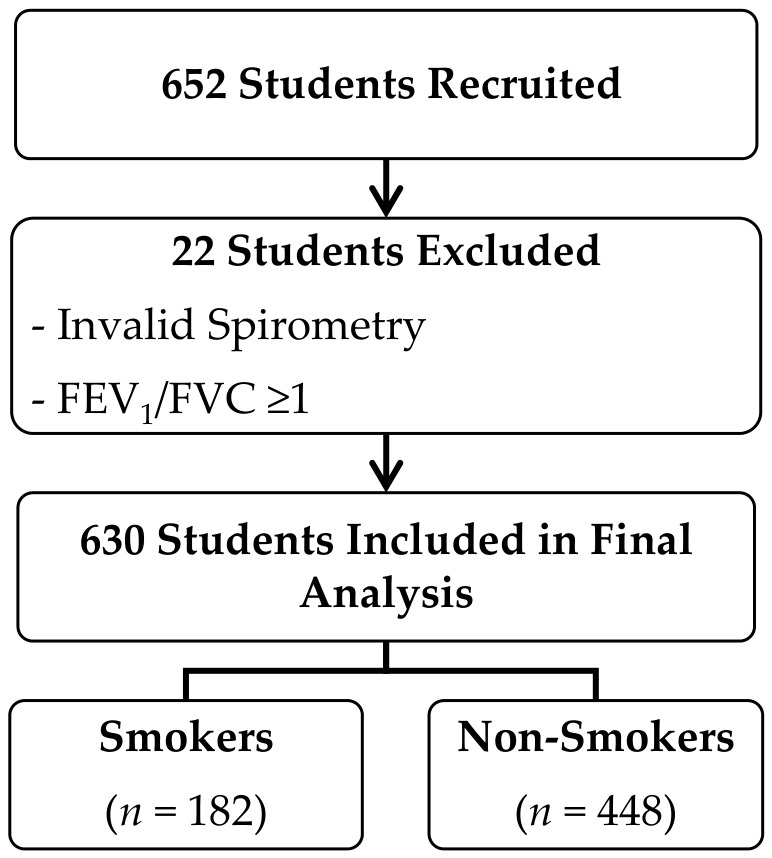
Flowchart of Participant Recruitment and Selection Process.

The study included students enrolled at Ajman University during the study period. Participants were recruited using a convenience sampling approach based on availability and willingness to participate. Initially, 652 students were recruited. Following spirometry quality assessment, 22 participants were excluded because of erroneous or invalid spirometry recordings, including physiologically implausible FEV_1_/FVC ratios ≥ 1.0. Consequently, 630 participants were included in the final analysis, corresponding to a response validity rate of 96.6%, as illustrated in the participant recruitment and selection flowchart (Figure 1).

Participants were categorized into smokers and non-smokers according to self-reported current smoking status. The smoker group included users of conventional cigarettes, electronic nicotine delivery systems (ENDS), shisha, and midwakh. Participants reporting the use of more than one nicotine product were classified as dual or multiple-product users within the smoker category. Non-smokers were defined as participants who reported no current use of nicotine-containing products.

Smoking exposure was assessed primarily according to current smoking status. Information regarding the type of nicotine product used was also collected; however, detailed quantitative exposure variables such as smoking duration, smoking intensity, frequency of use, and pack-year history were not consistently available for all participants and therefore were not included in the primary analysis.

Participants who had quit smoking within the previous three months were still classified as smokers, as residual physiological effects of smoking on pulmonary function may persist for weeks following smoking cessation [23]. Students with pre-existing respiratory diseases, including asthma and allergic bronchitis, or acute respiratory symptoms such as fever, cough, or sore throat, were excluded to minimize potential confounding effects on spirometric measurements.

A priori sample size estimation was performed using G*Power software (version 3.1.9.7) based on the primary outcome of detecting differences in FEV_1_/FVC ratios between smokers and non-smokers. Assuming an effect size of 0.29, a two-sided alpha level of 0.05, and 80% statistical power, the minimum required sample size was estimated to be 470 participants. Therefore, the final sample size of 630 participants was considered adequate to detect statistically significant differences in pulmonary function across study groups.

### 2.3. Data Collection Tools

Pulmonary function and anthropometric data were collected using the MIR SpiroLab 4 Spirometer (MIR, Rome, Italy), a calibrated weight–height scale, and disposable single-use mouthpieces with filters. Participants’ body weight and height were measured to calculate body mass index (BMI) as part of the anthropometric assessment. The spirometer was calibrated according to the manufacturer’s recommendations prior to testing sessions to ensure the accuracy and reliability of spirometric measurements, in accordance with the recommendations of the American Thoracic Society and European Respiratory Society spirometry standards. All spirometry tests were performed by trained members of the research team to ensure adherence to standardized testing procedures.

Spirometry procedures followed the standardized recommendations of the Standardization of Spirometry 2019 Update (ATS/ERS 2019) to ensure consistency, accuracy, and reproducibility [24,25]. Participants were instructed to empty their bladder and to avoid heavy meals, vigorous exercise, smoking, and caffeine for 2–3 h prior to testing. All spirometry measurements were performed with participants in the standing position to maintain procedural consistency throughout the study. A standing position was used to ensure optimal lung expansion and to facilitate maximal inspiratory and expiratory effort during spirometry. Participants were closely monitored throughout the procedure to ensure safety and to minimize the risk of presyncope or syncope.

Participant age, sex, height, and weight were recorded to calculate predicted lung function values using the Global Lung Function Initiative Reference Equations, which are internationally validated, derived from large multiethnic populations, and widely recommended for clinical and research comparability [26].

During testing, participants’ nostrils were clamped using a nose clip, and they were instructed to perform a full inspiration followed by a forceful and complete expiration into the mouthpiece until a sufficient plateau is observed on the graph. Each participant performed a minimum of three forced expiratory maneuvers. Only acceptable and repeatable maneuvers, defined by proper test initiation, maximal effort, and absence of artifacts such as coughing or premature termination, were included in the analysis according to American Thoracic Society/European Respiratory Society criteria. The highest recorded values of Forced Vital Capacity (FVC) and Forced Expiratory Volume in one second (FEV_1_) from acceptable trials were used for analysis, and the corresponding FEV_1_/FVC ratio was calculated. This standardized protocol ensured the reliability, reproducibility, and accuracy of spirometric measurements.

### 2.4. Statistical Analysis

All statistical analyses were performed using IBM SPSS Statistics Version 27 (IBM Corp., Armonk, NY, USA). Continuous variables were summarized as mean ± standard deviation (SD), while categorical variables were presented as frequencies and percentages. The distribution of continuous variables was assessed using the Shapiro–Wilk test and visual inspection of histograms. Although some spirometric variables demonstrated deviations from normality, parametric analyses were considered appropriate based on the study sample size and the robustness of General Linear Models to moderate departures from normality. Because several spirometric variables were not normally distributed (*p* < 0.05), non-parametric tests were applied to compare spirometric parameters between groups, including Forced Expiratory Volume in one second (FEV_1_), Forced Vital Capacity (FVC), and the FEV_1_/FVC ratio.

Smoking status, sex, and BMI were selected as primary analytical variables because they represent established physiological association with pulmonary function [27] and were directly aligned with the study objectives and available dataset variables. The association between sex and smoking status was assessed using a chi-square test of independence, and the odds ratio was calculated to estimate the strength of the association. Comparisons between two independent groups (e.g., smokers vs. non-smokers and males vs. females) were conducted using the Mann–Whitney U test, while differences across multiple groups, including BMI categories, were evaluated using the Kruskal–Wallis H test. As these non-parametric tests rely on ranked data, group differences were interpreted based on mean ranks rather than raw values. Post hoc pairwise comparisons were not performed. A *p*-value < 0.05 was considered statistically significant.

Additional potential confounding variables, including passive smoking exposure, physical activity, environmental pollution exposure, socioeconomic status, prior respiratory infection history, and undiagnosed respiratory disease were not systematically assessed and therefore could not be adjusted for in the present analysis.

Records with incomplete or invalid spirometry measurements were excluded prior to analysis; therefore, no missing spirometric data were included in the final dataset. The assumptions underlying the statistical analyses were assessed before analysis. Normality of continuous variables was evaluated using the Shapiro–Wilk test, which demonstrated non-normal distribution for several spirometric variables (*p* < 0.05), supporting the use of non-parametric statistical methods. Post hoc pairwise comparisons following the Kruskal–Wallis H test were not performed because the primary objective was to evaluate overall group differences rather than multiple subgroup comparisons, and some subgroup sample sizes were relatively unequal, which may reduce the reliability of extensive pairwise testing.

### 2.5. Ethics

The study protocol was reviewed and approved by the Ethics Committee of Ajman University, reference number: (IRB No. M-F-H-21-August) (31 August 2023), and was carried out in accordance with the ethical principles outlined in the Declaration of Helsinki. Participation in the study was entirely voluntary. Prior to enrollment, all participants received a clear explanation of the study objectives, procedures, and the voluntary nature of participation. Verbal informed consent was obtained from each participant before data collection. In accordance with the determination of the ethics committee, written consent was not required because the study was non-interventional and posed minimal risk to participants. No incentives were provided for participation, and no personally identifiable information was collected. All data were anonymized prior to analysis to ensure strict confidentiality and protection of participants’ privacy.

## 3. Results

### 3.1. Demographics

A total of 630 students from Ajman University were included in the final analysis. The study population consisted of 381 males (60.5%) and 249 females (39.5%). The majority of participants were aged 20–22 years (44.9%), followed by 17–19 years (38.9%), while smaller proportions were aged 23–25 years (14.1%), 26–30 years (1.4%), and over 30 years (0.6%). Most participants were of Caucasian background (94.3%), with smaller proportions from Pakistan (2.4%), South India (1.7%), North India (1.1%), and African descent (0.5%). In terms of nutritional status, nearly half of the participants (48.7%) had a healthy body mass index (BMI), while 29.3% were overweight, 10.9% had obesity, 6.5% had severe obesity, and 4.6% were underweight, as seen below in Table 1.

Regarding smoking status, 448 students (71.1%) were classified as non-smokers, whereas 182 students (28.9%) were smokers. Smoking was more prevalent among males, with 147 male smokers (23.3%) compared with 35 female smokers (5.6%), as seen below in Table 1. Among the 182 smokers, different types of nicotine products were reported, including vaping devices (*n* = 160, 87.9%), cigarettes (*n* = 62, 34.1%), shisha (*n* = 40, 22%), and midwakh (*n* = 37, 20.3%), with some students reporting the use of more than one product type. These findings indicate that vaping represents the most reported form of nicotine consumption within the study population, as seen below in Table 2.

### 3.2. Association Between Sex and Smoking Status

A chi-square test of independence was conducted to examine the association between sex and smoking status. Among female participants, 214 were non-smokers and 35 were smokers, representing a smoking prevalence of 14.1%. In contrast, among male participants, 234 were non-smokers and 147 were smokers, corresponding to a smoking prevalence of 38.6%. The chi-square analysis demonstrated a statistically significant association between sex and smoking status (χ^2^(1) = 42.91, *p* < 0.001). Further analysis indicated that males were significantly more likely to smoke than females, with an odds ratio (OR) of 3.84 (95% CI: 2.53–5.82), suggesting that the odds of smoking among males were nearly four times those among females. Logistic regression analysis was also performed to further evaluate this relationship. The results showed an intercept (constant) of −1.81 (*p* < 0.001) and a coefficient for sex (Male = 1, Female = 0) of 1.35 (*p* < 0.001). The positive coefficient for sex indicates that being male is associated with a higher likelihood of smoking, and the statistically significant *p*-value confirms that sex is a significant predictor of smoking behavior in this sample. The odds ratio derived from the regression coefficient, exp (1.35), is approximately 3.86, indicating that males are about 3.9 times more likely to smoke compared with females.

### 3.3. Stratified Analysis of Lung Function by Smoking Status

**Figure 2:**
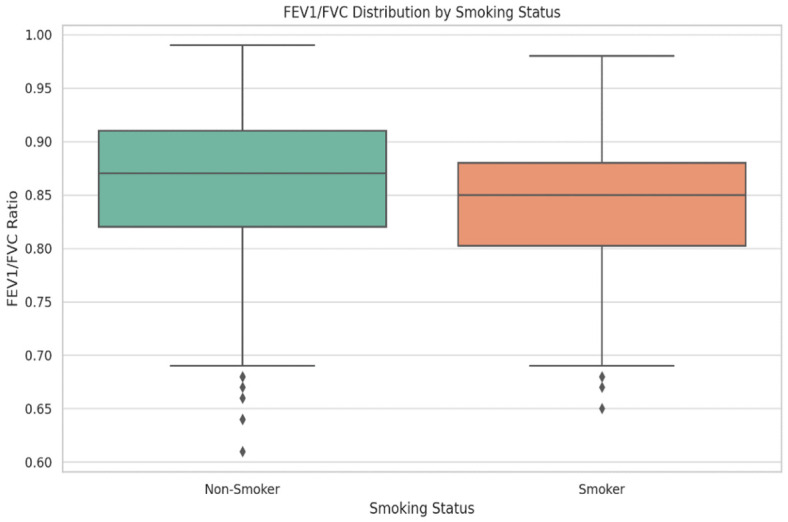
FEV_1_/FVC Ratio Distribution by Smoking Status.

**Figure 3:**
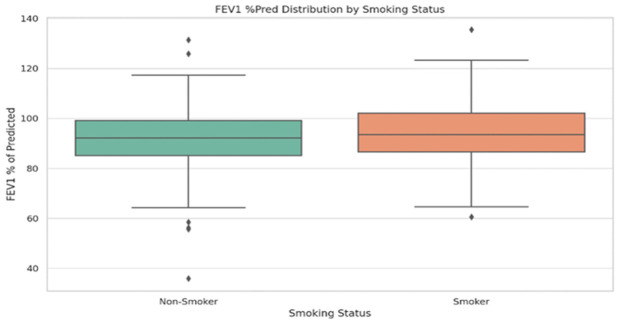
FEV_1_ % Predicted Distribution by Smoking Status.

**Figure 4:**
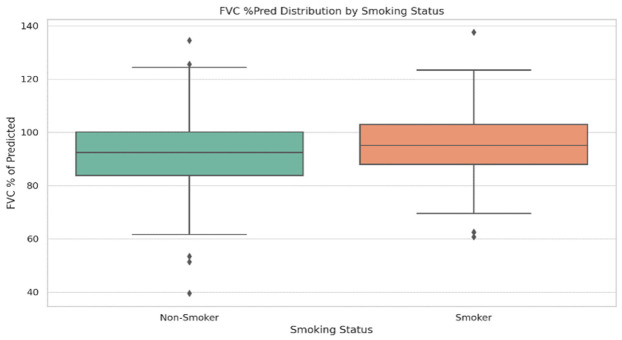
FVC % Predicted Distribution by Smoking Status.

The study included 630 participants and examined differences in lung function according to smoking status. Compared with non-smoking students, smokers demonstrated a statistically significant lower FEV_1_/FVC ratio (0.84 ± 0.07) than non-smokers (0.86 ± 0.07) (*p* < 0.001) (Table 3, Figure 2), suggesting the presence of early airflow changes among smokers. In contrast, smokers showed slightly higher predicted percentages for forced expiratory volume in one second (FEV_1_ % predicted: 94.01 ± 12.33 vs. 91.45 ± 11.51; *p* = 0.048) and forced vital capacity (FVC % predicted: 95.71 ± 12.17 vs. 91.65 ± 12.47; *p* < 0.01) compared with non-smokers, reflecting the overall preservation of lung volumes within the normal predicted range in both groups (Table 3, Figure 3 and Figure 4).

These findings indicate that the reduction in the FEV_1_/FVC ratio among smokers occurred despite relatively preserved predicted lung volumes, suggesting that airflow limitation may develop before marked reductions in FEV_1_ or FVC become clinically apparent. Table 3 demonstrates that the difference in the FEV_1_/FVC ratio was the most consistent and clinically relevant finding between smokers and non-smokers, supporting its value as an early marker of subclinical airway obstruction in young adults. Figure 2 further illustrates the lower distribution of FEV_1_/FVC ratios among smokers compared with non-smokers, reinforcing the presence of early expiratory airflow changes associated with nicotine exposure.

### 3.4. Stratified Analysis of Lung Function by Sex

**Figure 5:**
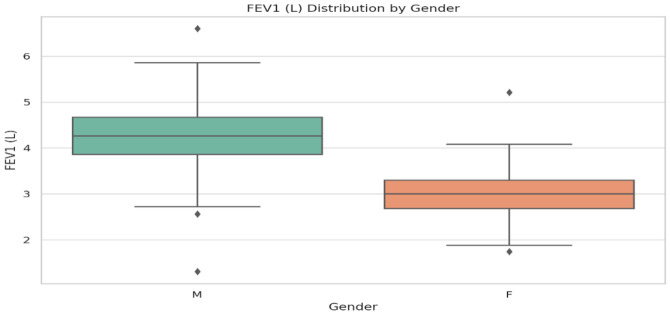
FEV_1_(L) Mean Distribution by Sex.

**Figure 6:**
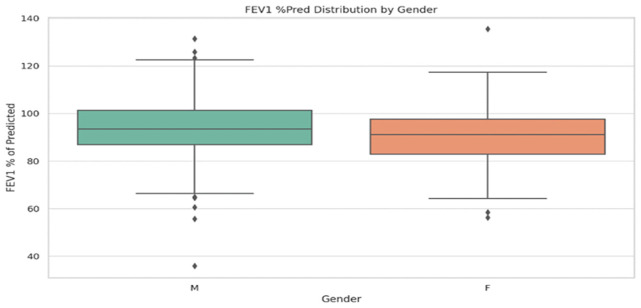
FEV_1_ Predicted Mean Distribution by Sex.

**Figure 7:**
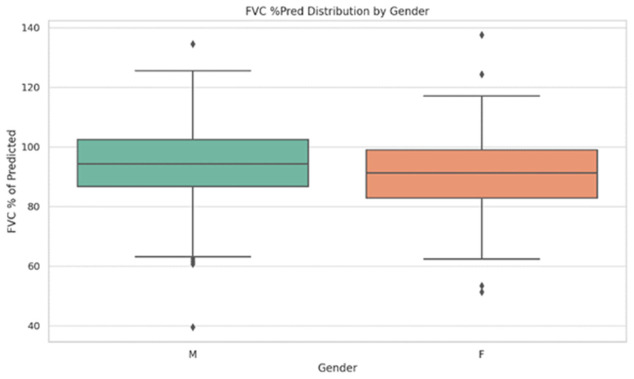
FVC Predicted Mean Distribution by Sex.

**Figure 8:**
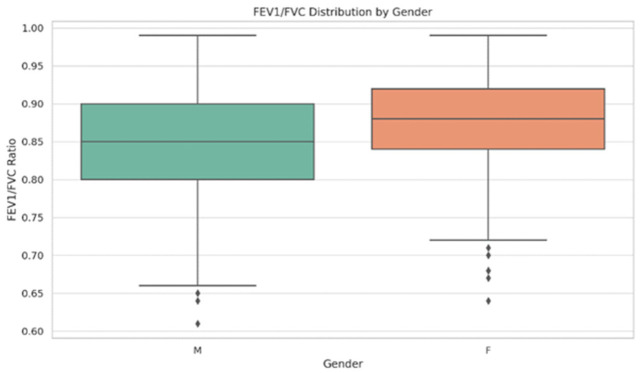
FEV_1_/FVC Ratio Predicted Distribution by Sex.

Spirometric parameters differed between males and females, irrespective of smoking status (Table 4, Figure 5, Figure 6, Figure 7 and Figure 8). Males had a statistically significant higher predicted lung volumes than females, with mean FEV_1_ values of 4.25 ± 0.64 L (93.6 ± 11.7% predicted) compared with 2.98 ± 0.47 L (90.1 ± 11.6% predicted) in females (U = 4848.5, *p* < 0.001). Similarly, FVC was higher in males (94.3 ± 12.6% predicted) than in females (90.5 ± 12.1% predicted) (U = 38,833, *p* < 0.001). In contrast, females exhibited a higher FEV_1_/FVC ratio than males (0.88 ± 0.06 vs. 0.85 ± 0.07) (U = 60,047.5, *p* < 0.001).

The sex-based differences presented in Table 4 show that males had significantly higher predicted FEV_1_ and FVC values, whereas females demonstrated consistently higher FEV_1_/FVC ratios. This pattern likely reflects normal physiological differences in lung size and airway dimensions between sexes rather than pathological impairment. In addition, smoking prevalence was substantially higher among males, which may partly contribute to the lower airflow ratios observed in male participants. These findings highlight the importance of interpreting spirometry values in the context of both biological sex and smoking behavior.

### 3.5. Stratified Analysis of Lung Function by Smoking Status and Sex

**Figure 9:**
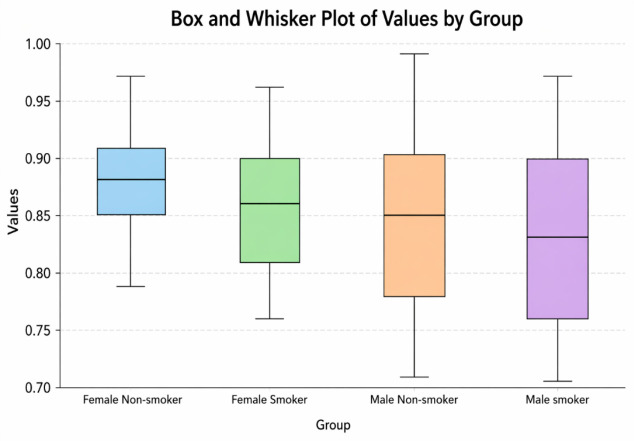
Distribution of FEV_1_/FVC Ratio by Smoking Status and Sex. Box-and-whisker plot showing FEV_1_/FVC ratios across four groups: female non-smokers, female smokers, male non-smokers, and male smokers. The central line represents the median, the box indicates the interquartile range (IQR), and the whiskers represent the range of observed values. Male smokers exhibited the lowest FEV_1_/FVC ratios, whereas female non-smokers demonstrated the highest values.

Further stratified analysis by smoking status and sex revealed distinct patterns in pulmonary function (Table 5, Figure 9). The highest FEV_1_/FVC ratio was observed among female non-smokers (0.88 ± 0.06), followed by male non-smokers (0.85 ± 0.07) (*p* < 0.001). Among smokers, female smokers maintained a relatively higher ratio (0.86 ± 0.05), whereas male smokers exhibited the lowest ratio (0.83 ± 0.07).

Predicted lung volumes also varied between groups. Female non-smokers had lower predicted lung volumes (FEV_1_: 89.58 ± 11.22%; FVC: 89.78 ± 11.83%) compared with male non-smokers (FEV_1_: 93.15 ± 11.54%; FVC: 93.36 ± 12.83%). Among smokers, males demonstrated slightly higher predicted values (FEV_1_: 94.26 ± 12.08%; FVC: 95.88 ± 12.04%) compared with female smokers (FEV_1_: 92.96 ± 13.49%; FVC: 94.96 ± 12.85%).

**Figure 10:**
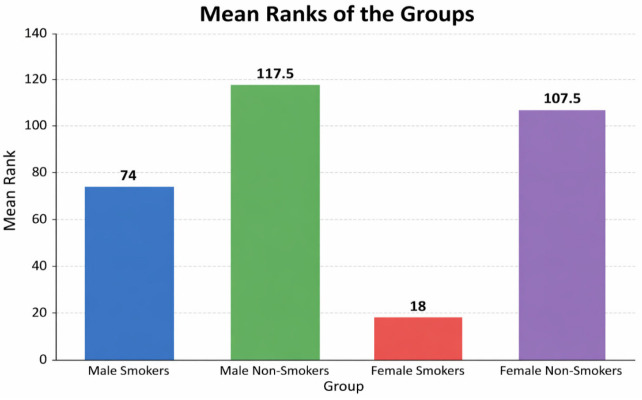
Mean ranks of FEV_1_ across study groups based on sex and smoking status.

A Kruskal–Wallis Test was conducted to evaluate differences in FEV_1_ values among the four study groups defined by sex and smoking status (Figure 10). The analysis revealed a highly statistically significant difference in FEV_1_ across the groups (H = 364.93, *p* = 8.74 × 10^−79^) (*p* < 0.001). Because the Kruskal–Wallis test is based on ranked data rather than raw means, group comparisons were interpreted using mean ranks. Female smokers had the lowest mean rank (18), indicating the lowest FEV_1_ values, followed by male smokers (mean rank = 74). In contrast, female non-smokers (mean rank = 107.5) and male non-smokers (mean rank = 117.5) demonstrated substantially higher ranks, with male non-smokers showing the highest FEV_1_ values overall. These findings suggest significant differences in lung function among the groups, with smoking associated with lower FEV_1_ values, particularly among female smokers, while non-smokers exhibited comparatively better pulmonary function.

### 3.6. Stratified Analysis of Lung Function by BMI

**Figure 11:**
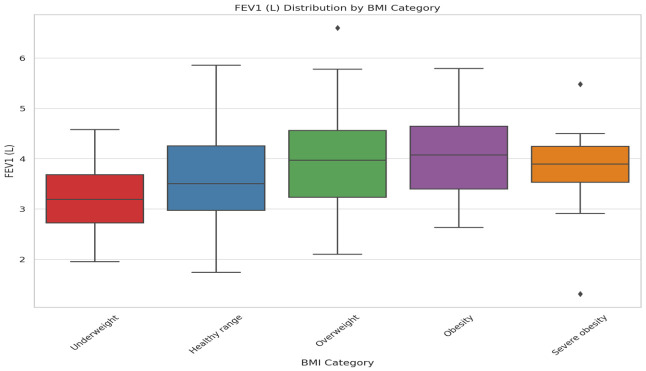
FEV_1_(L) Mean Distribution by BMI Category.

**Figure 12:**
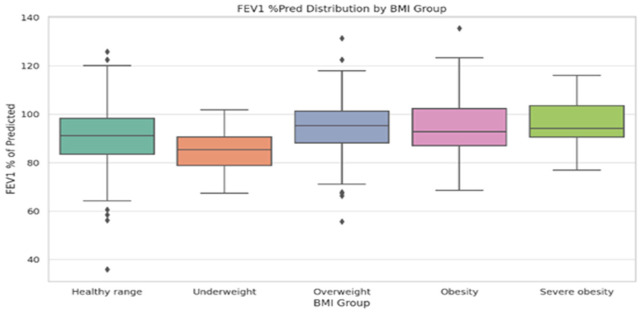
FEV_1_ Predicted Distribution by BMI Category.

**Figure 13:**
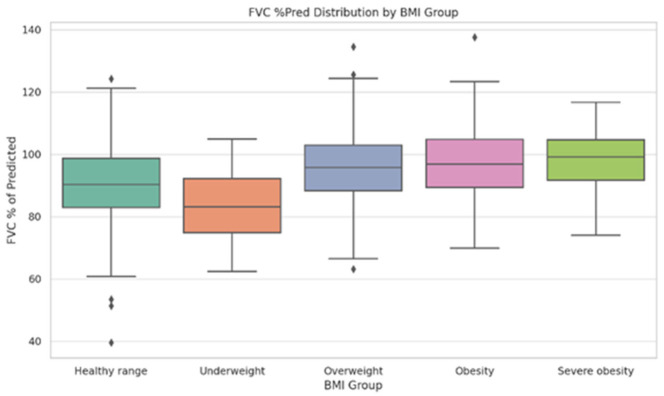
FVC Ratio Predicted distribution by BMI category.

**Figure 14:**
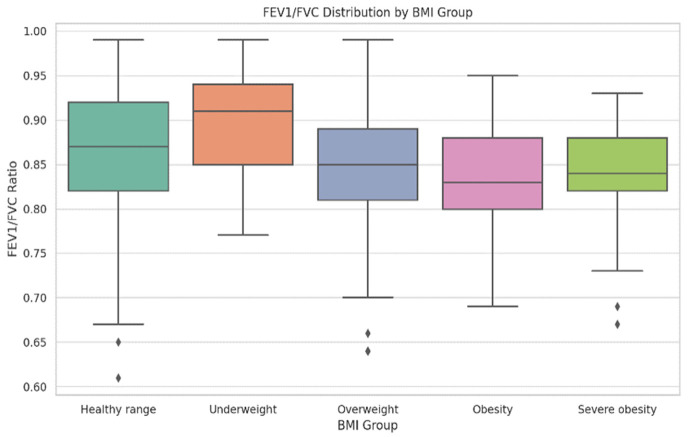
FEV_1_/FVC Ratio Predicted distribution by BMI category.

Lung function differed across BMI categories in the study population (Table 6, Figure 11, Figure 12, Figure 13 and Figure 14). A Kruskal–Wallis H test showed significant differences in FEV_1_ (L) among BMI groups (H(4) = 41.61, *p* < 0.001). Underweight participants had the lowest predicted FEV_1_ (85.0 ± 9.8) and FVC (83.9 ± 11.5) and the highest FEV_1_/FVC ratio (0.89 ± 0.06). Participants with a healthy BMI had intermediate FEV_1_ (90.6 ± 12.1) and FVC (90.5 ± 12.6) values and a FEV_1_/FVC ratio of 0.87 ± 0.07. Overweight individuals had higher FEV_1_ (94.3 ± 11.2) and FVC (95.3 ± 11.3) and a lower FEV_1_/FVC ratio (0.85 ± 0.06). Obese participants showed FEV_1_ of 94.2 ± 11.6, FVC of 97.5 ± 13.0, and FEV_1_/FVC ratio of 0.83 ± 0.06, while severely obese participants had FEV_1_ of 96.4 ± 9.8, FVC of 98.2 ± 9.0, and FEV_1_/FVC ratio of 0.84 ± 0.06.

As shown in Table 6, increasing BMI was associated with progressively higher predicted FEV_1_ and FVC values, while the FEV_1_/FVC ratio showed a gradual decline from underweight to obese categories. Underweight participants demonstrated the lowest lung volumes but the highest FEV_1_/FVC ratios, whereas overweight and obese participants showed higher lung volumes with lower airflow ratios. This pattern suggests that increased BMI may mask early airflow limitation when interpretation relies only on lung volumes, emphasizing the importance of evaluating the FEV_1_/FVC ratio alongside BMI during spirometric assessment.

## 4. Discussion

The present study provides important insights into the influence of smoking status, sex, and body mass index on lung function among young adults. Although the study population largely consisted of apparently healthy university students, significant variations in spirometric parameters were observed across different subgroups. Smoking was associated with a reduction in the FEV_1_/FVC ratio, suggesting the presence of early airflow limitation. The findings demonstrate a substantial decline in lung function among smokers compared with non-smokers. However, considering that spirometry may lack sufficient sensitivity to detect early small airway dysfunction or subtle airway alterations, particularly in young smokers, the incorporation of advanced diagnostic modalities such as impulse oscillometry (IOS) and inert gas washout testing would considerably strengthen the study. These techniques enable the detection of early smoking-induced airway abnormalities that may remain undetected through conventional spirometric assessment.

In addition, clear sex-based differences were identified, with males demonstrating higher lung volumes and females exhibiting relatively higher FEV_1_/FVC ratios. Furthermore, BMI showed a distinct relationship with pulmonary function, whereby increasing BMI was associated with higher lung volumes but lower FEV_1_/FVC ratios, highlighting early airflow changes in overweight and obese students. These findings collectively indicate that multiple factors, including behavioral and physiological characteristics, interact to influence respiratory function, even at a young age and in the absence of overt disease.

It should be noted that smokers were intentionally oversampled during the spirometry phase of our two-phase study at Ajman University to enhance the detection of early airflow changes [22]. Consequently, the proportion of smokers in the analyzed sample was higher than in the initial survey, which should be considered when interpreting the generalizability of these findings.

### 4.1. Smoking and Airflow Limitation: FEV_1_/FVC as an Early Marker of Obstruction

The present study demonstrated that smoking is associated with reduced expiratory airflow among university students. Smokers exhibited a statistically significant (*p* < 0.001) reduction in the FEV_1_/FVC ratio compared with non-smokers, suggesting possible early airflow limitation that may precede measurable reductions in lung volumes. This finding is consistent with the well-established physiological effects of tobacco exposure, including airway inflammation, oxidative stress, and small airway narrowing, which collectively impair expiratory flow before detectable declines in lung volumes occur [28]. Notably, smokers maintained normal predicted values of forced expiratory volume in one second (FEV_1_) and forced vital capacity (FVC) while demonstrating a reduced FEV_1_/FVC ratio, highlighting the sensitivity of this index in detecting early airflow obstruction, even in the absence of clinical symptoms [29]. These findings suggest that subtle changes in airflow may already be present among young adult smokers, underscoring the importance of early identification of respiratory impairment in populations with emerging nicotine exposure. Additionally, the preservation of lung volumes (FEV_1_ % predicted and FVC % predicted) in both groups may reflect the influence of demographic factors such as sex and height.

The high prevalence of vaping observed in this study is noteworthy, as the majority of smokers (≈88%) reported using electronic nicotine delivery systems. This finding reflects the increasing popularity of e-cigarettes among young adults. Although often perceived as safer alternatives to conventional cigarettes, emerging evidence suggests that e-cigarettes may have comparable adverse respiratory effects, including airway inflammation and oxidative stress [30,31]. The findings of this study raise concern that smoking, including vaping, may contribute to early subclinical pulmonary dysfunction in young populations.

### 4.2. Sex-Based Differences in Spirometry Results

Sex-based analysis revealed significant differences in spirometric parameters between males and females, irrespective of smoking status, highlighting the importance of interpreting spirometry results in the context of biological sex to ensure accurate clinical assessment [32,33]. In this study, females consistently demonstrated higher FEV_1_/FVC ratios than males across all smoking categories, reflecting known physiological and anatomical differences in airway size, lung mechanics, and airflow dynamics. These differences may also be influenced by sex-specific factors such as nicotine metabolism, hormonal modulation of inflammatory responses, and lung dimensions [34].

Although females may maintain relatively preserved airflow in the early stages of nicotine exposure, some evidence suggests they are more susceptible than males to long-term smoking-related lung injury [35,36]. Notably, in this study, female smokers retained higher FEV_1_/FVC ratios compared with male smokers, whereas male smokers exhibited the lowest FEV_1_/FVC ratios, indicating greater early susceptibility to airflow limitation. In contrast, predicted lung volumes (FEV_1_ % and FVC %) varied modestly between sexes, with males generally demonstrating higher values than females across both smoking and non-smoking categories. These differences likely reflect underlying anthropometric factors such as height and lung size rather than pathological changes. Interestingly, male smokers in our cohort exhibited normal predicted lung volumes (FEV_1_: 94.3 ± 12.1%; FVC: 95.9 ± 12.0%) despite a reduction in the FEV_1_/FVC ratio. This pattern suggests that relying solely on predicted lung volume percentages may fail to detect early airflow abnormalities, emphasizing the need for clinicians to interpret spirometry results in conjunction with airflow indices and smoking history when evaluating young smokers.

Further analysis using a Kruskal–Wallis test demonstrated significant differences in FEV_1_ across groups defined by sex and smoking status, supporting the observed variability in lung function. The rank-based comparisons indicated that smokers, particularly female smokers, had lower FEV_1_ values, whereas non-smokers exhibited higher values, with male non-smokers demonstrating the highest lung function overall. These findings reinforce the impact of smoking on pulmonary function even in young adults and highlight that early functional changes may be detectable before overt reductions in predicted lung volumes occur. Overall, although lung volumes remained within normal predicted ranges in this young population, the observed smoking-related reduction in airflow indices; particularly among male smokers; may indicate early subclinical airway impairment preceding measurable declines in lung volumes [34]. Taken together, these results emphasize the value of early spirometric screening in young smokers for the detection of subclinical airflow limitation and the opportunity for timely preventive interventions.

### 4.3. Limitations of Predicted Spirometry Percentages in Young Smokers

Predicted spirometric values are commonly used to assess lung function; however, they may have limitations in detecting early functional impairment in smokers. In our study, smokers maintained relatively normal predicted values, despite demonstrating reduced airflow ratios. Because predicted values incorporate age, sex, and height, they may mask subtle functional abnormalities in individuals with preserved lung volumes but impaired airflow [37]. This phenomenon may be particularly relevant in young smokers, who may compensate through increased lung expansion or hyperinflation and often remain asymptomatic during the early stages of disease development [37]. Consequently, reliance solely on predicted spirometry percentages may lead to missed opportunities for early detection of smoking-related airway disease [38].

### 4.4. Influence of Body Mass Index on Lung Function

The present study demonstrated a clear association between BMI and lung function among the participants. Underweight individuals exhibited the lowest predicted FEV_1_ and FVC values, yet maintained the highest FEV_1_/FVC ratio, indicating that while lung volumes were reduced, airflow remained relatively preserved. This observation is consistent with findings from a large Korean study, which similarly reported that underweight adults had lower lung volumes but preserved FEV_1_/FVC ratios, suggesting that reduced body mass is associated with decreased lung capacity without early airflow limitation [39]. Participants with a healthy BMI showed moderate lung volumes and slightly lower FEV_1_/FVC ratios, consistent with normal pulmonary function. In contrast, overweight and obese individuals demonstrated higher FEV_1_ and FVC values but progressively lower FEV_1_/FVC ratios, suggesting the presence of early airflow limitation despite increased lung volumes. These findings align with previous research indicating that obesity alters lung mechanics, reduces chest wall compliance, and contributes to airflow limitation [40]. This effect is likely explained by increased chest wall and abdominal mass in individuals with higher BMI, which can restrict lung expansion and reduce expiratory airflow, even in the absence of overt respiratory disease.

The pattern observed in severely obese participants further stresses the impact of elevated BMI on pulmonary function, highlighting the importance of monitoring lung health even in young and apparently healthy populations. Collectively, these results suggest that increased BMI may mask early airflow limitation through preserved or elevated lung volumes, while subtle reductions in the FEV_1_/FVC ratio may represent early indicator sign of pulmonary compromise.

Furthermore, the transition from underweight to a healthy BMI was associated with improved predicted lung volumes, likely reflecting enhanced respiratory muscle strength and overall physiological capacity. However, excessive adiposity may negatively affect pulmonary mechanics and expiratory airflow. These observations emphasize the importance of considering BMI when interpreting spirometry results, as failure to do so may lead to misclassification of pulmonary function abnormalities, consistent with previous population-based evidence on the association between body habitus and lung function [41].

### 4.5. Quality of Spirometry Data

Ensuring high-quality spirometry measurements is essential for reliable assessment of pulmonary function. In the present study, approximately 3.4% of spirometry recordings were graded as unsatisfactory and excluded from the primary analysis. Inclusion of these lower-quality recordings slightly increased mean FVC and FEV_1_ values but did not alter the overall trends observed in the study. Nevertheless, the use of suboptimal spirometry data may lead to overestimation of lung function parameters and potentially obscure true differences between groups. Adherence to standardized spirometry procedures is therefore critical for accurate data interpretation [24].

### 4.6. Comparison with Previous Studies

The findings of the present study are consistent with previous research conducted among university and young populations. A study from the University of Lahore reported that smoking was associated with significant reductions in lung function indices, particularly FEV_1_ and FEV_1_/FVC ratios among smokers [42]. Similarly, investigations involving university students have demonstrated that even individuals with relatively mild smoking habits exhibited respiratory symptoms and measurable reductions in lung function, highlighting the early impact of smoking on respiratory health [43,44]. Furthermore, studies conducted among university athletes have shown that smoking is associated with decreased pulmonary performance despite high levels of physical activity, emphasizing that smoking-related respiratory impairment can occur even in otherwise healthy young adults [45].

### 4.7. Public Health Implications

Despite the relatively short duration of smoking exposure in this young population, a notable proportion of smokers already exhibited subclinical pulmonary dysfunction, evidenced by reductions in the FEV_1_/FVC ratio. Previous studies, including those involving patients with Chronic Obstructive Pulmonary Disease, have demonstrated that smoking initiation at a younger age is associated with increased respiratory symptoms and poorer long-term outcomes [30]. These findings emphasize the critical need for early smoking prevention and cessation programs targeting young adults in university populations. Interventions should address conventional cigarettes, e-cigarettes, and other nicotine delivery systems such as shisha to preserve respiratory health and prevent progression to overt disease. Furthermore, the results of this study support the use of spirometry as an early screening tool to detect subtle airflow abnormalities in young smokers, with careful consideration of the influence of sex and BMI on lung function when interpreting results. Promoting healthy lifestyle behaviors, alongside early detection of airflow changes through spirometry, may help prevent the progression to long-term respiratory disease.

## 5. Study Strengths and Limitations

This study has several important strengths. First, it included a relatively large sample size (*n* = 630) for a spirometry-based investigation among university students, which improves the reliability and statistical power of the findings. Second, pulmonary function testing was conducted using standardized procedures in accordance with the guidelines of the American Thoracic Society and the European Respiratory Society, ensuring consistency and quality of spirometry measurements. Lung function values were interpreted using the Global Lung Function Initiative reference equations, which are internationally validated and derived from large multiethnic populations, thereby enhancing the comparability of the results.

Another strength of the study is the inclusion of sex-specific and body mass index (BMI) analyses, allowing a more comprehensive evaluation of factors that may influence lung function. Importantly, the study focused on young adults, a population that is often underrepresented in smoking-related pulmonary research despite increasing use of nicotine products in this age group.

Several limitations should be considered when interpreting the findings of this study. First, the cross-sectional design limits the ability to establish a causal relationship between nicotine use and changes in lung function. Longitudinal studies are required to determine whether the observed airflow differences progress over time more pulmonary impairment and lead to clinically significant respiratory disease.

Second, the use of convenience sampling may introduce selection bias and limit the generalizability of the findings. In addition, the study relied on self-reported smoking status, which may introduce reporting bias, particularly in settings where smoking carries social or cultural stigma. Additionally, smoking exposure was not quantified in terms of duration of smoking or pack-years, limiting the ability to assess potential dose–response relationships between nicotine exposure and lung function impairment.

Third, post-bronchodilator spirometry was not performed; therefore, reversible and fixed airflow limitation could not be differentiated. Although smokers demonstrated lower FEV_1_/FVC ratio values compared with non-smokers, these values remained largely within the normal range, which may limit the clinical interpretation of early airflow changes.

Fourth, spirometry is also an effort-dependent test, and the accuracy of its results may be influenced by participant cooperation and operator technique, which may introduce measurement variability despite adherence to standardized testing protocols. Although strict quality criteria were applied, measurement variability cannot be completely excluded. However, despite the measures taken to minimize confounding bias through participant selection and exclusion criteria, it is not possible to completely exclude possible residual confounding, as the results of pulmonary function measurements could have been affected by other factors that were not measured, such as undiagnosed respiratory diseases, levels of physical activity, environmental exposures (including air pollution and passive smoking), and the duration and intensity of nicotine use, along with other lifestyle-related variables and socioeconomic status. The unmeasured factors subject the analysis to further bias.

Additionally, although different nicotine products were reported, the study did not perform a detailed stratified analysis comparing exclusive electronic nicotine delivery systems (ENDS) users, cigarette smokers, midwakh users, shisha users, and poly-users, all of whom may exhibit distinct exposure patterns and physiological effects. Because many participants reported using more than one nicotine product, the present study primarily evaluated overall smoking exposure rather than the isolated effects of specific nicotine product types. Consequently, the limited differentiation between nicotine product groups may have reduced the ability to detect product-specific respiratory effects due to overlapping nicotine product use.

Moreover, the results from the study may lack external credibility, as it involves students from only one university. Thus, it might not be relevant to all young adults who are not currently attending university, to those living in other geographical regions, or to those from different socioeconomic backgrounds.

Finally, Mid-expiratory flow indices, such as FEF_25–75_, were not analyzed. However, according to recommendations from the European Respiratory Society and the American Thoracic Society, interpretation of pulmonary function tests should primarily rely on core spirometric indices (FEV_1_, FVC, and the FEV_1_/FVC ratio), as mid-expiratory measures exhibit greater biological variability and limited diagnostic reliability [46].

## 6. Conclusions

This is a regional study that provides data on spirometric lung function of students from Ajman University in the United Arab Emirates who consume a variety of nicotine products, such as cigarettes, shisha, midwakh and ENDS (electronic nicotine delivery systems). The study demonstrates that the use of nicotine was associated with measurable changes in pulmonary function in university students.

Smoking, sex and BMI are all factors that affect lung function even in young and apparently healthy people. The results emphasize the need for early screening, careful interpretation of spirometry results, and the implementation of public health preventive measures that target smoking and obesity to maintain respiratory health in the youth.

From the stratified analysis, the FEV_1_/FVC ratio was the lowest in the male smokers group and the highest in the female non-smokers group among the cohorts. Lung function parameters also differed greatly between the BMI groups. Underweight participants had the lowest FEV_1_ and FVC values and the highest FEV_1_/FVC ratio, while the overweight and obese participants had higher lung volumes and lower FEV_1_/FVC ratios. The results indicate that the FEV_1_/FVC ratio is a practical spirometric tool to detect variations in airflow among smokers and non-smokers in the young adult population.

The results add to the growing body of research on the respiratory consequences of nicotine use in young adults and could help support future research and public health efforts aimed at preventing and reducing nicotine product use among university students. Smoking cessation programs should not only address cigarette smoking but should also address other forms of nicotine delivery systems such as vaping, midwakh, and shisha as well.

## Figures and Tables

**Table 1:** Demographic Characteristics of the Study Population.

Parameter	Sub-Category	*n* (%) ^b,c^
Sex	Male	381 (60.5)
	Female	249 (39.5)
Age Group (years)	17–19	245 (38.9)
	20–22	283 (44.9)
	23–25	89 (14.1)
	26–30	9 (1.4)
	30+	4 (0.6)
Ethnic Group	Caucasian	594 (94.3)
	Pakistan	15 (2.4)
	South Indian	11 (1.7)
	North Indian	7 (1.1)
	African Descent	3 (0.5)
BMI Group ^a^	Healthy range	306 (48.7)
	Overweight	185 (29.3)
	Obesity	69 (10.9)
	Severe obesity	41 (6.5)
	Underweight	29 (4.6)
Smoking Status ^d^	Smoker	182 (28.9)
	Non-Smoker	448 (71.1)
Smoking Status by Sex	Male	147 (23.3%)
	Female	35 (5.6%)
Non-Smoking Status by Sex	Male	234 (37.14%)
	Female	214 (33.96%)

^a^ BMI, body mass index; ^b^ Values are presented as mean ± standard deviation (SD) or *n* (%), as appropriate. ^c^ The initial sample included 652 students; 630 participants met the spirometry acceptability criteria and were included in the final analysis. ^d^ Smoking status includes current smokers and participants who had quit smoking within the previous three months.

**Table 2:** Types of Nicotine Products Used Among Smokers.

Type of Nicotine Use ^b^	Frequency ^a^	(%) of Total Smokers ^a^
Vaping Devices	160	87.9
Cigarettes	62	34.1
Shisha	40	22
Midwakh	37	20.3
Total Smokers	182 (includes mixed smoking of more than one product type)	

^a^ Values are presented as *n* (%). ^b^ Smoking status includes current smokers and participants who had quit smoking within the previous three months.

**Table 3:** Comparison of Lung Function Parameters Stratified by Smoking Status *.

Parameter	Smokers ^b,c^ (Mean ± SD)	Non-Smokers ^b^ (Mean ± SD)	U Statistic	*p*-Value
FEV_1_/FVC Ratio ^a,d^	0.84 ± 0.07	0.86 ± 0.07	31,696	<0.001
FEV_1_ % Predicted	94.01 ± 12.33	91.45 ± 11.51	44,855	0.048
FVC % Predicted	95.71 ± 12.17	91.65 ± 12.47	47,333.5	<0.01

* Data is expressed as mean ± standard deviation (SD). Comparisons between Smokers and Non-Smokers were performed using the Mann–Whitney U test. A *p*-value < 0.05 was considered statistically significant. ^a^ FEV_1_, forced expiratory volume in one second; FVC, forced vital capacity. ^b^ Values are presented as mean ± standard deviation (SD) or *n* (%), as appropriate. ^c^ Smoking status includes current smokers and participants who had quit smoking within the previous three months. ^d^ Predicted spirometry values were calculated using GLI reference equations.

**Table 4:** Comparison of Lung Function Parameters Stratified by Sex *.

Parameter	Female (Mean ± SD)	Male (Mean ± SD)	U Statistic	*p*-Value
FEV_1_/FVC Ratio ^a,b^	0.88 ± 0.06	0.85 ± 0.07	60,047.5	<0.001
FEV_1_ % Predicted	90.1 ± 11.6	93.6 ± 11.7	38,958.5	<0.001
FVC % Predicted	90.5 ± 12.1	94.3 ± 12.6	38,833	<0.001

* Data is reported as mean ± standard deviation (SD). Comparisons between Males and Females were performed using the Mann–Whitney U test. A *p*-value < 0.05 was considered statistically significant. ^a^ FEV_1_, forced expiratory volume in one second; FVC, forced vital capacity; ^b^ Predicted spirometry values were calculated using GLI reference equations.

**Table 5:** Comparison of Lung Function Parameters Stratified by Smoking Status and Sex ^c^.

Category	FEV_1_/FVC ^a,b^	FEV_1_ % Predicted ^b^	FVC % Predicted ^b^
Female Non-smoker	0.88 ± 0.06	89.58 ± 11.22	89.78 ± 11.83
Female Smoker	0.86 ± 0.05	92.96 ± 13.49	94.96 ± 12.85
Male Non-smoker	0.85 ± 0.07	93.15 ± 11.54	93.36 ± 12.83
Male smoker	0.83 ± 0.07	94.26 ± 12.08	95.88 ± 12.04

^a^ FEV_1_, forced expiratory volume in one second; FVC, forced vital capacity. ^b^ Values are presented as mean ± standard deviation (SD). ^c^ Predicted spirometry values were calculated using GLI reference equations.

**Table 6:** Lung Function Parameters by BMI Categories ^c,d,e,f^.

Category	FEV_1_ % Predicted ^a^ (Mean ± SD)	FVC % Predicted ^b^ (Mean ± SD)	FEV_1_/FVC (Mean ± SD)
Underweight	85.0 ± 9.8	83.9 ± 11.5	0.89 ± 0.06
Healthy range	90.6 ± 12.1	90.5 ± 12.6	0.87 ± 0.07
Overweight	94.3 ± 11.2	95.3 ± 11.3	0.85 ± 0.06
Obesity	94.2 ± 11.6	97.5 ± 13.0	0.83 ± 0.06
Severe obesity	96.4 ± 9.8	98.2 ± 9.0	0.84 ± 0.06
Kruskal–Wallis H test results	29.59	52	36.91
*p*-value	<0.001	<0.001	<0.001

^a^ FEV_1_, forced expiratory volume in one second; ^b^ FVC, forced vital capacity; ^c^ BMI, body mass index; ^d^ Values are presented as mean ± standard deviation (SD) or *n* (%), as appropriate. ^e^ Predicted spirometry values were calculated using GLI reference equations. ^f^ Comparisons between groups were performed using the Mann–Whitney U test or Kruskal–Wallis H test, as appropriate. A *p*-value < 0.05 was considered statistically significant.

## Data Availability

The datasets generated and/or analyzed during the current study are available from the corresponding author upon reasonable request.
